# Characterization and Genome Analysis of the *Delftia lacustris* Strain LzhVag01 Isolated from Vaginal Discharge

**DOI:** 10.1007/s00284-024-03758-x

**Published:** 2024-06-19

**Authors:** Li Zhang, Xin Zhang, Huihui Bai, Ting Li, Zhan Zhang, Xiaonan Zong, Xiang Shang, Zhaohui Liu, Linyuan Fan

**Affiliations:** Beijing Obstetrics and Gynecology Hospital, Beijing Maternal and Child Health Care Hospital, Capital Medical University, Beijing, 100026 China

## Abstract

*Delftia* has been separated from freshwater, sludge, and soil and has emerged as a novel opportunistic pathogen in the female vagina. However, the genomic characteristics, pathogenicity, and biotechnological properties still need to be comprehensively investigated. In this study, a *Delftia* strain was isolated from the vaginal discharge of a 43-year-old female with histologically confirmed cervical intraepithelial neoplasm (CIN III), followed by whole-genome sequencing. Phylogenetic analysis and average nucleotide identity (ANI) analysis demonstrated that it belongs to *Delftia lacustris*, named *D. lacustris* strain LzhVag01. LzhVag01 was sensitive to β-lactams, macrolides, and tetracyclines but exhibited resistance to lincoamines, nitroimidazoles, aminoglycosides, and fluoroquinolones. Its genome is a single, circular chromosome of 6,740,460 bp with an average GC content of 66.59%. Whole-genome analysis identified 16 antibiotic resistance-related genes, which match the antimicrobial susceptibility profile of this strain, and 11 potential virulence genes. These pathogenic factors may contribute to its colonization in the vaginal environment and its adaptation and accelerate the progression of cervical cancer. This study sequenced and characterized the whole-genome of *Delftia lacustris* isolated from vaginal discharge, which provides investigators and clinicians with valuable insights into this uncommon species.

## Introduction

*Delftia* is a gram-negative, aerobic, motile, non-spore-forming, and non-fermenting bacillus genus belonging to the *Comamonadaceae* [1]. The genus is divided into six species: *D. acidovorans* isolated from soil [2], *D. tsuruhatensis* isolated from activated sludge [3], *D. lacustris* isolated from freshwater [4], *D. litopenaei* isolated from a freshwater shrimp culture pond [5], *D. deserti* isolated from a desert soil [6] and *D. rhizosphaerae* recovered from the rhizosphere of *Cistus ladanifer* [7]. Although *Delftia* was rarely associated with human infections since it was first isolated from a patient with catheter-related infection caused by *D. tsuruhatensis* in 2011 [8], isolation of this organism from patients with immunological deficiency has increased in recent years. Cases include endocarditis [9], ocular infection [10], urinary tract infection, empyema, renal injury, hepatocellular carcinoma and renal infarction [11].

The vaginal microbiota is a community mainly composed of *lactobacilli* and coexisting with various microorganisms in the female vagina [12]. These microbiota are mutually restrictive and balanced to maintain vaginal health. Increasing evidence shows that genital tract dysbiosis and/or specific bacteria and cytokines might have an active role in pathogenic infection and also in the development of cervical intra-epithelial neoplasia (CIN), which seriously affects women’s health [13]. *Delftia* are widely overlooked bacteria in the vaginal microbiota. Wu et al. found that *Delftia* genus is enriched in the low-grade squamous intraepithelial lesions (LSIL) and high-grade squamous intraepithelial lesions (HSIL) groups and may be a potential biomarker of CIN progression [14]. A growing number of studies have shown that *Delftia* was associated with high discomfort or pain following vaginal intercourse [15] and unexplained recurrent pregnancy loss (RPL) [16], which seriously affect both women’s physical and mental health.

According to a report by the World Health Organization in 2020, cervical cancer contributes to the greatest degree for female mortality, claiming ~ 341,831 lives annually [17]. Considering that *Delftia* might have a serious impact on obstetrics and gynecology diseases, particularly in the progression of cervical cancer, it is urgently necessary to isolate and analyze its genomic characteristics. To our knowledge, currently, there is no report of *Delftia* species isolated from the female vagina, which greatly limits our understanding of its genomic characteristics, drug resistance characteristics, virulence factors, and pathogenic mechanisms.

In this study, a novel strain of *Delftia—Delftia lacustris* strain LzhVag01—was isolated from the vaginal discharge of a 43-years-old female with histologically verified cervical intraepithelial neoplasia III (CIN III) at Beijing Obstetrics and Gynecology Hospital, Capital Medical University, Beijing Maternal and Child Health Care Hospital in China. To the best of our knowledge, *Delftia* has never been isolated from human vaginal discharge before this work. To gain a deeper insights into LzhVag01, we conducted whole-genome sequencing and performed extensive comparative genomics analysis of LzhVag01 with other sequenced *Delftia* strains to elucidate the genome divergence of these microorganisms at the genus level. Additionally, we undertook a comparative genomic analysis of pathogenic bacteria associated with human infections to elucidate their genomic characteristics and potential mechanisms of drug resistance and pathogenicity. This study is critical to understanding the pathogenic mechanism of *Delftia lacstris* and the diagnosis and treatment of obstetrics and gynecology diseases.

## Materials and Methods

### Bacterial Isolation and Identification

The LzhVag01wild-type strain was isolated in 2023 from the vaginal discharge of a 43-year-old female at Beijing Obstetrics and Gynecology Hospital, Capital Medical University, Beijing Maternal and Child Health Care Hospital in China. Histologically verified CIN III, thinprep cytologic test (TCT) revealed inflammation, Human Papillomavirus (HPV) Test indicated HPV16 positive, and vaginal microecology evaluation showed abnormal vaginal microbiota. The patient denies having multiple sexual partners, suffering from sexually transmitted diseases (STDs), or coming into contact with contaminated water or places in the wild.

Vaginal secretions were collected using a cotton swab, immersed in 1 mL of PBS solution, and following a tenfold dilution, 100 μL of sample was inoculated onto Luria-Bertani broth (LB) agar plate (Sigma-Aldrich), then incubated at 37 ℃ for 24 h. Picked colonies were purified, streaked 3 times, then inoculated into LB medium (Sigma-Aldrich), grown overnight at 37 ℃ with shaking at 180 rpm, and immediately stored as a glycerol stock. Gram staining was performed as previously described to confirm the morphology and structure of the bacterium [12]. The isolate was initially identified by 16S rDNA sequencing and subsequently confirmed by phylogenetic analysis of LzhVag01 with *Delftia* species based on whole-genome sequences. The maximum-likelihood tree was bootstrapped 100 in PhyML v20151210 [18]. Further, the identity of the strain was verified by calculating the average nucleotide identity (ANI) values using FastANI [19].

### Antimicrobial Susceptibility Testing

Antimicrobial susceptibility was determined according to the Clinical and Laboratory Standards Institute (CLSI, 2021) Minimum Inhibitory Concentration (MIC) Interpretive Standards for ‘‘Enterobacterales’’ and ‘‘non-Enterobacterales’’ using the broth microdilution method, and the resistance breakpoints for each drug were defined as CLSI M100-Ed31 guidelines [20]. *E. coli* ATCC 25922 was used as the reference strain for quality control. All drugs were purchased from the National Institute for the Control of Pharmaceutical and Biological Products (NICPBP).

### Genome Sequencing, Assembly, and Annotation

The genomic DNA was extracted using the HiPure Bacterial DNA Kit (Guangzhou Magen Biotechnology Co., Ltd), followed by purification with Ampure XP beads (Beckman), and quantified using NanoDrop One spectrophotometer (NanoDrop Technologies) and Qubit 3.0 Fluorometer (Invitrogen). The genomic library were constructed using VAHTS Universal Plus DNA Library Prep Kit (Nanjing Vazyme Biotech Co.,Ltd) for the Illumina sequencing and Ligation Sequencing Kit (SQK-LSK109 and SQK-LSK104/114, Oxford Nanopore Technologies) for Nanopore sequencing, then sequenced on the Illumina NovaSeq 6000 (Illumina) and Nanopore PromethION P48 (Oxford Nanopore Technologies) platforms, respectively.

For the genome assembly, the raw data of Illumina sequencing was filtered using fastp v0.23.0[21] to obtain clean data. The nanopore sequencing reads were assembled by Canu v1.7 [22] and Flye v2.6 [23], then correct the genome with Pilon v1.22 [24] using previous Illumina clean data. Potential open reading frames (ORFs) were predicted using Prodigal v2.6.3 [25], transfer RNA (tRNA) genes were predicted with tRNAscan-SE v2.0 [26], and ribosome RNA (rRNA) genes were predicted using Barrnap [27]. The coding genes were annotated with the National Center for Biotechnology Information (NCBI) nr database by Diamond v0.8.15 (e < 1e-05) [28]. The genomic context of LzhVag01 was visualized through Circos v0.69 [29]. The core and pan-genes were clustered by the CD-HIT rapid clustering of similar proteins software (v 4.8.1) with a threshold of 50% pairwise identity and 0.7 length difference cutoff in amino acid [30]. Venn diagrams and gene dilution curves were produced using the perl module SVG and R package boxplot, respectively. The comparative genomic circular map was constructed by BRIG v0.95 [31]. The protein-encoding genes were classified using the eggNOG database [32] and predicted using the eggNOG-Mapper v2 [33]. The antimicrobial resistance (AMR) genes and virulence factors were predicted using the Comprehensive Antibiotic Resistance Database (CARD) and the Virulence Factor Database (VFDB) with Diamond [34], respectively. The data were deposited in the GenBank database under the following accession numbers: BioProject, PRJNA1049760; BioSample, SAMN38710906; GenBank, CP141536.

## Results

### Identification of *D. lacustris* Strain LzhVag01

**Fig. 1:**
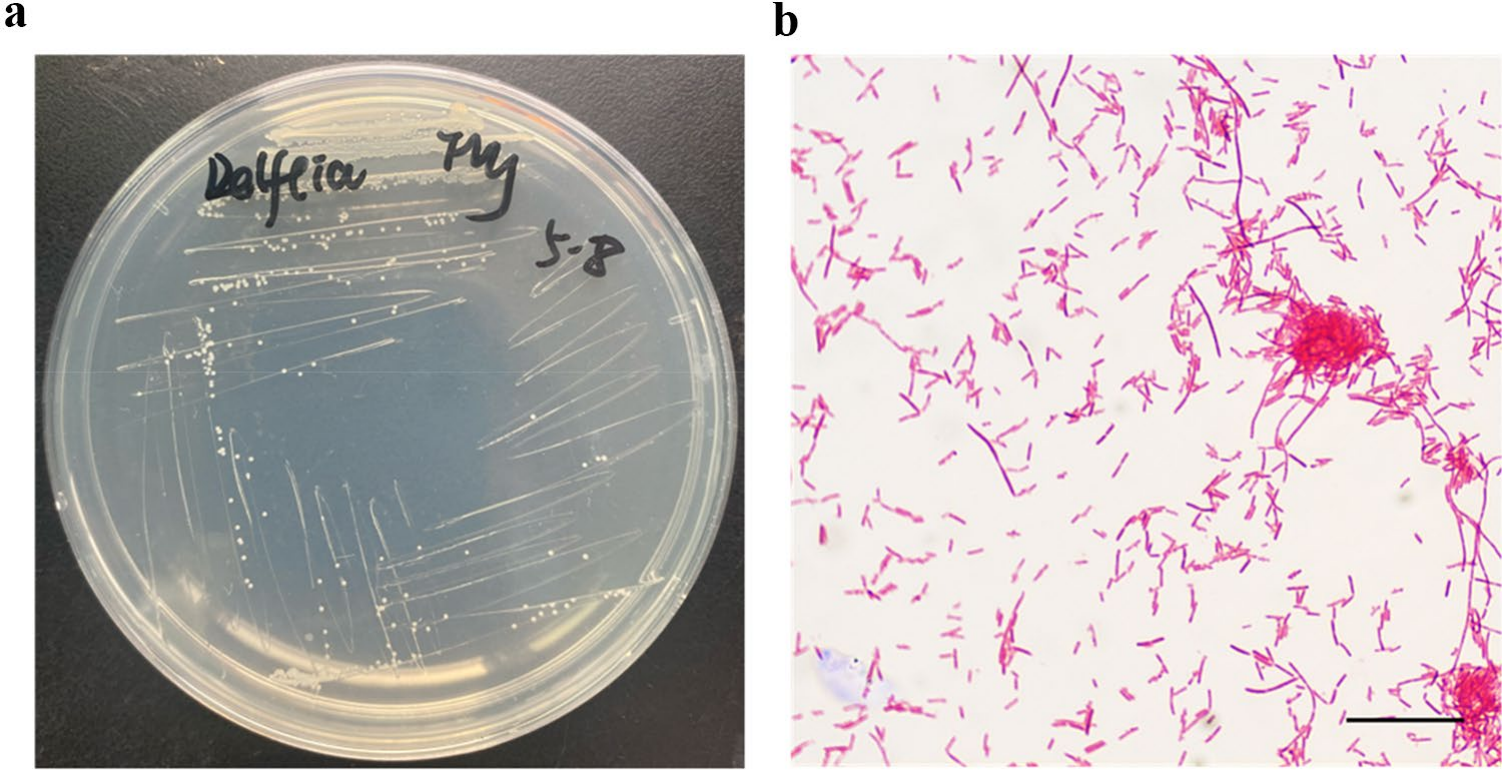
Morphological characteristics of LzhVag01. LzhVag01 displayed white colonies on the LB agar plate (**a**) and gram-negative rods by Gram staining (**b**). Scale bar = 25 μm

*Delftia* strain LzhVag01 was isolated from the vaginal discharge on an LB agar plate incubated at 37 ℃ for 24 h. The colonies of LzhVag01 were white-cream, round, smooth, and convex with approximate diameters of 1–3 mm (Fig. 1a). Microscopy revealed long, straight, Gram-negative rods with diameters ranging from 1 to 8 μm and the length of some cells reached 28 μm (Fig. 1b). 16S rDNA sequencing showed that LzhVag01 belonged to the genus *Delftia.*

**Fig. 2:**
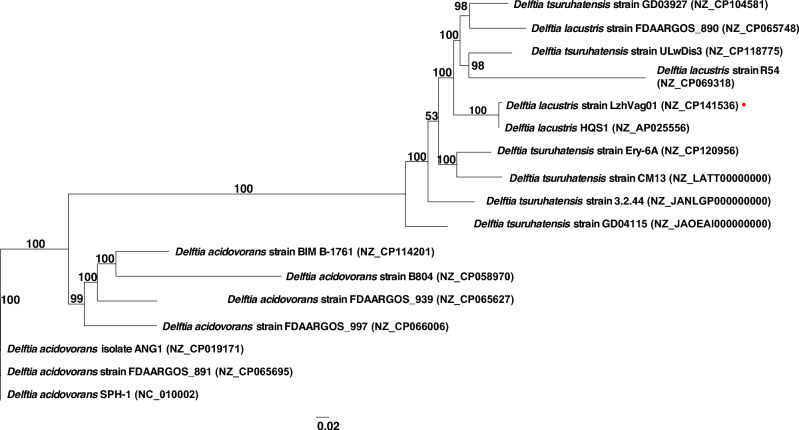
Phylogenetic relationships of *D. lacustris* strain LzhVag01 with other type strains of *Delftia* species based on whole-genome sequences. LzhVag01 was highlighted with a red dot. GenBank accession numbers were listed in parentheses following the species names

Whole-genome phylogeny and comparisons have the power to evaluate the evolutionary relationship and phylogenetic position with high resolution. A maximum-likelihood (ML) tree was constructed based on whole-genome shared by 17 *Delftia* species genomes (Fig. 2). Phylogenetic trees showed that LzhVag01 exhibited the closest evolutionary relationship with *D. lacustris* HQS1 (NZ_AP025556).

ANI was defined as the mean nucleotide identity of orthologous gene pairs shared between two microbial genomes, and FastANI was performed to calculate the average nucleotide identity (ANI) values. The ANI values between LzhVag01 and the above two *D. lacustris* strains were 99.86% (HQS1) and 98.32% (FDAARGOS_890), respectively, which exceeded the threshold of 95–96% for species circumscription [19]. Therefore, the LzhVag01 was grouped into the species *D. lacustris.*

### General Features of *D. lacustris* Strain LzhVag01 Genome

**Fig. 3:**
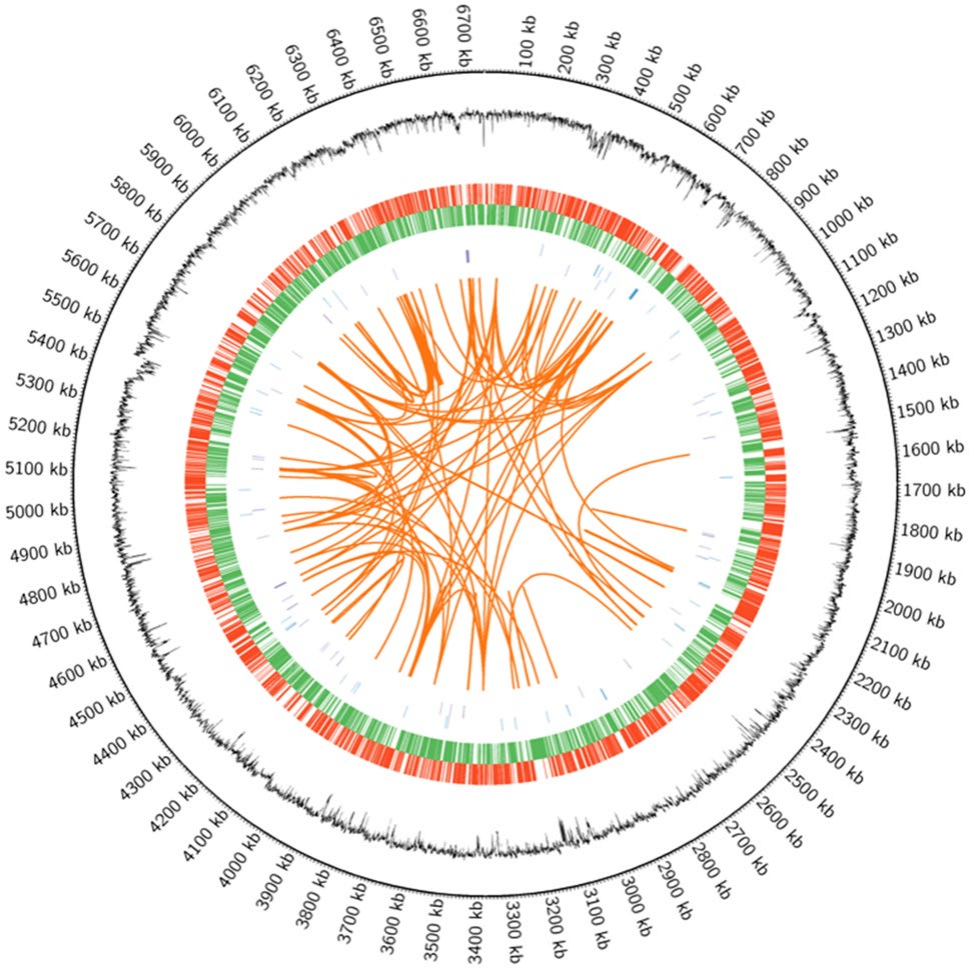
Circular representation of the genome of *D. lacustris* strain LzhVag01. Counting from the outside toward the center: circle 1 shows the genome size of LzhVag01, each scale represents 5 kb; circle 2 represents GC content; circles 3–4 indicates positive (red) and negative (green) strands genes of the genome; circles 5 displays the ncRNA on positive strands (blue); circles 6 refer to the ncRNA on negative strands (purple); circles 7 represent long segment repeat sequence information within genome (orange)

Whole-genome Nanopore PromethION P48 produced more than 8,626,560 reads and 137,314 contigs ranging from 2000 to 259,648 bp in size (N50 contig sizes of 20,177 bp). Following processed Illumina reads mapped onto the primary assembly to correct the consensus of Nanopore, the LzhVag01 genome was assembled into one circular chromosome of 6,740,460 bp in size with an average GC content of 66.59%, approximately 89.76% (6,050,142 bp) of nucleotides were predicted to be in protein-coding regions, and there was no evidence of plasmids. Prodigal v2.6.3 annotation revealed that the genome comprised 6040 Protein-coding genes. A total of 150 RNA genes were predicted, including 79 tRNAs, 15 rRNAs, and 56 other ncRNA. Among the predicted genes, 78.41% (4,736 genes) were assigned to COG categories. The genome circle diagram of LzhVag01 is shown in Fig. 3.

### Pan-Genome analysis of *Delftia* species

To characterize the genetic diversity of the *Delftia* species, pan-genome represented by 17 *Delftia* genomes, LzhVag01 (NZ_CP141536.1), ANG1 (NZ_CP019171.1), SPH-1 (NC_010002), B804 (NZ_CP058970.1), BIM B-1761 (NZ_CP114201.1), FDAARGOS_891 (NZ_CP065695.1), FDAARGOS_909 (NZ_CP065668.1), FDAARGOS_939 (NZ_CP065627.1), FDAARGOS_997 (NZ_CP066006.1), HQS1 (NZ_AP025556.1), FDAARGOS_890 (NZ_CP065748.1), R54 (NZ_CP069318.1), CM13 (NZ_LATT00000000.1), Ery-6A (NZ_CP120956.1), TR1180 (NZ_CP045291.1), GD03927 (NZ_CP104581.1) and ULwDis3 (NZ_CP118775.1), from NCBI genome database were estimated. The genome sizes ranged from 6.351 Mb (FDAARGOS_939) to 7.196 Mb (CM13). These strains were obtained from human, sea water, sink and soil, exhibiting niche diversity.

**Fig. 4:**
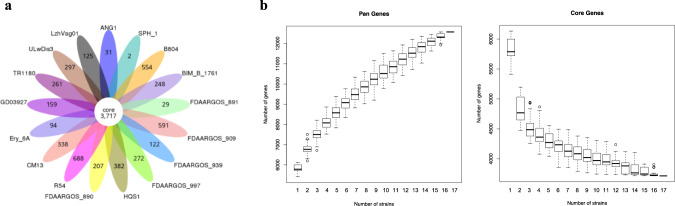
Pan-genome analysis of *Delftia* species. **a** Flower plot of 17 *Delftia* species genomes showing the gene content of core genome (flower center) and strain-specific genes (flower petals). **b** The cumulative curves for the core and pan-genome of *Delftia* species. The curves showed the downward trend of the core gene families and the upward trend of the pan-gene families with the increase in the number of genomes

A total of 12,577 pan-genome gene families were identified (Fig. 4a). Among these, 3717 (29.5%) represented the core genome, and the remaining 8860 (70.4%) represented the accessory genome (4651, 37.0%) and strain-specific genes (4209, 33.4%). The small size of the core genome in *Delftia* species results in an expansive accessory genome and strain-specific genes. The number of strain-specific genes across different genomes exhibited a wide distribution, varying from 2 in *D. acidovorans* SPH-1 to 688 in *D. lacustris* strain R54, suggest that the diverse genetic evolution in different *Delftia* strains. Out of the 125 strain-specific genes identified in the LzhVag01 genome, the majority of the genes annotated by COG were associated with transcription regulation, replication, recombination, and repair. However, the functions of most of these genes (59.2%) remain unknown. Therefore, further analysis was warranted to elucidate the specific functions of these genes in LzhVag01. The pan-genome accumulation curve showed it had not reached saturation, even though there are more than 17 species (Fig. 4b). This suggests that the *Delftia* pan-genome was open and evolving, indicating species of this genus can colonize different environments and have various ways of exchanging genetic material [35].

### Comparative Analysis with Strains LzhVag01 and Other Related Genomes

To further clarify the genome structure and function of strains isolated from human and environmental, three *Delftia* species genomes available from GenBank, *D. tsuruhatensis strain* TR1180 (NZ_CP045291), *D. tsuruhatensis* strain ULwDis3 (NZ_CP118775) and *D. acidovorans* isolate ANG1 (NZ_CP019171) were used for comparative genome analysis with *D. lacustris* strain LzhVag01. The general features of these genomes are given in Table 1. The genomic sizes of these strains exhibited minor variations.

**Table 1:** General features of *D. lacustris* strain LzhVag01 and other related genomes

	*D. lacustris* strain LzhVag01	*D. tsuruhatensis strain* TR1180	*D. tsuruhatensis strain* ULwDis3	*D. acidovorans isolate ANG1*
Source	*Homo sapiens*	*Homo sapiens*	Water	Soil
Genome size	6,740,460	6,711,018	6,944,081	6,556,728
G + C percentage	66.59%	66.52%	66.52%	66.57%
CDS number	6040	6021	6308	5764

**Fig. 5:**
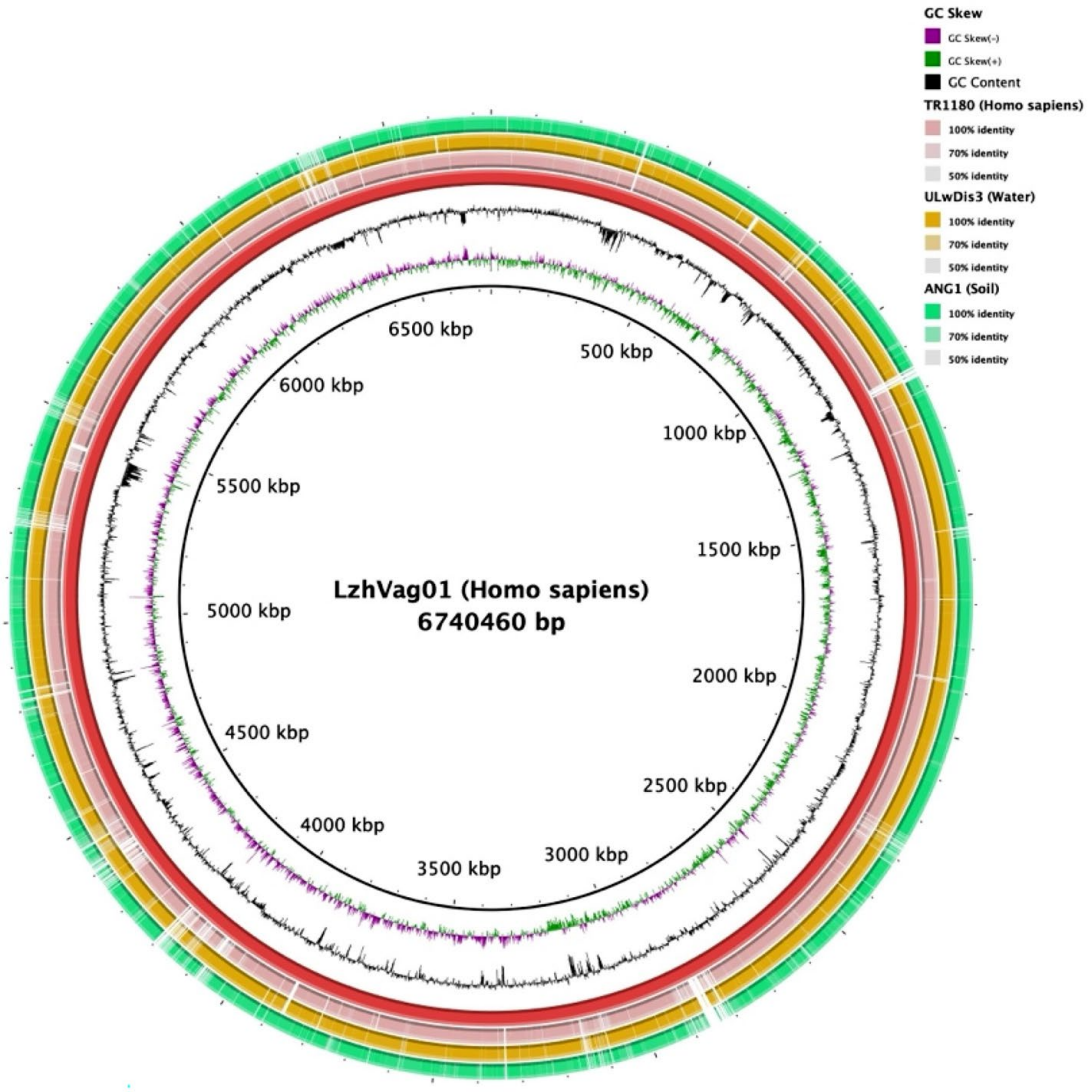
Circular representation of the *D. lacustris* strain LzhVag01 genome and comparative genomics analysis with other *Delftia* strains generated by BRIG. Counting from the outside toward the center: circles 1–4 refer to regions of *D. acidovorans* ANG1 (green), *D. tsuruhatensis* ULwDis3 (yellow), *D. tsuruhatensis strain* TR1180 (pink) and *D. lacustris* strain LzhVag01 (red), where empty regions indicate parts without similar hits between them; circles 5 and 6 represent GC content and GC skew of LzhVag01, respectively

A comparative genome circle was generated by BRIG, the visual inspection of the circular alignment of theses genomes highlights that three of the genomes were similar to the alignment reference genome of strain LzhVag01. However, it’s visually apparent in the figure that, in comparison to LzhVag01, the other three strains exhibit more than 10 regions of absent in their genomes (Fig. 5). These regions encompass several enzymes crucial for metabolism, including genes encoding formate dehydrogenase, catalase C, and succinyl-coa synthetase. Additionally, enzymes involved in transposition, recombination, and DNA damage repair were also identified.

**Fig. 6:**
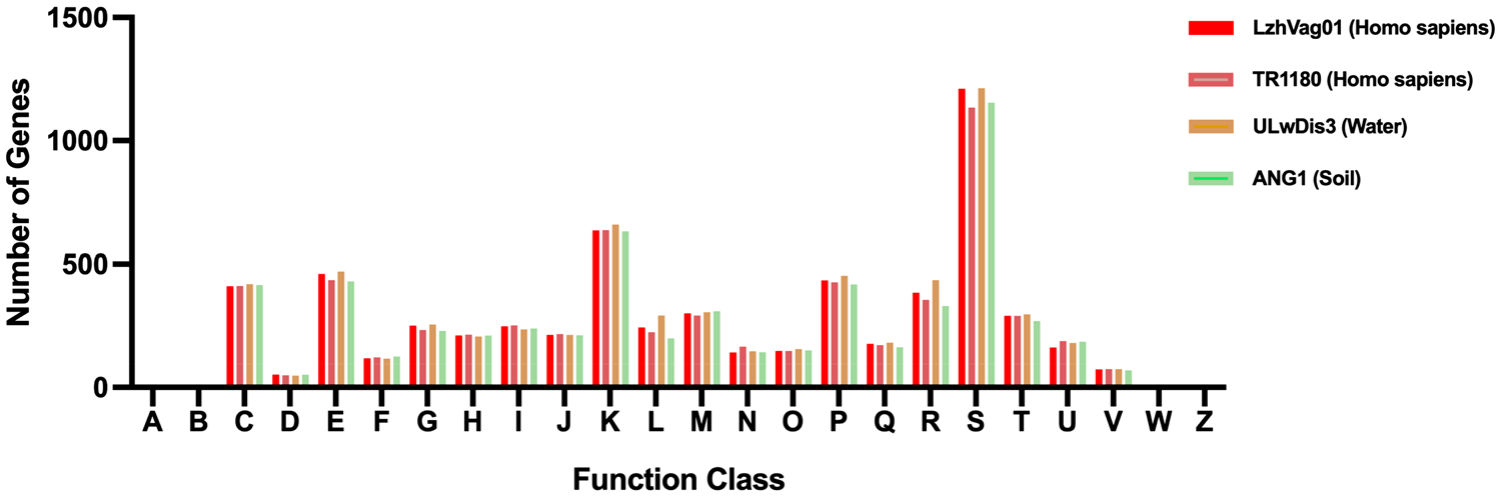
COG categories of the genes in each strain. COG functional categories are described as follows: A, RNA processing and modification; B, Chromatin structure and dynamics; C, energy production and conversion; D, cell cycle control, cell division, chromosome partitioning; E, amino acid transport and metabolism; F, nucleotide transport and metabolism; G, carbohydrate transport and metabolism; H, coenzyme transport and metabolism; I, lipid transport and metabolism; J, translation, ribosomal structure, and biogenesis; K, transcription; L, replication, recombination, and repair; M, cell wall/membrane/envelope biogenesis; N, cell motility; O, posttranslational modification, protein turnover, chaperones; P, inorganic ion transport and metabolism; Q, secondary metabolite biosynthesis, transport, and catabolism; R, general function prediction only; S, function unknown; T, signal transduction mechanisms; U, intracellular trafficking, secretion, and vesicular transport; V, defense mechanisms; W, Extracellular structures; Z, Cytoskeleton

To obtain a deeper understanding of the functional enrichment of each component in these genome, clusters of orthologous groups of proteins (COG) analysis was performed by eggNOG-Mapper v2 to categorize the function of LzhVag01 and other strains. The gene families were assigned to 24 COG functional categories, function of most of the genes was the unknown (S), genes involved in transcription (K) represented the most abundant functional category, apart from the amino acid transport and metabolism (E), indicating that most of the genes were associated with housekeeping functions, regardless of whether the strain was isolated from human or the environment. Other common gene classes were inorganic ion transport and metabolism (P), energy production and conversion (C), and signal transduction mechanisms (T). COG-based analysis demonstrated that the genes of these four genomes show a similar distribution trend in terms of COG category (Fig. 6).

### Antibiotic Susceptibilities and Associated Genes

Emergence of antibiotic resistant pathogenic bacteria poses a serious challenge to the treatment of diseases. *Delftia* species have intrinsic resistance to aminoglycoside antibiotics [36]. Our antibiotic susceptibility profiling results demonstrated that LzhVag01 exhibited a multidrug-resistant (MDR) phenotype (Table 2). In addition to aminoglycoside (gentamicin) resistance, LzhVag01 was resistant to lincosamides (clindamycin), nitroimidazoles (metronidazole) and fluoroquinolones (levofloxacin), but it was sensitive to most β-lactams (cefoxitin sodium, ceftazidime, ceftriaxone), macrolides (azithromycin) and tetracyclines (minocycline). These results suggested that as an emerging opportunistic human pathogen, LzhVag01 was evolving to be resistant to a greater range of antibiotics. Interestingly, *D. tsuruhatensis strain* TR1180, isolated from the sputum of a patient with respiratory failure, and LzhVag01 exhibited nearly identical resistance phenotypes. This suggests that the two strains of *Delftia* from human hosts likely share a common origin and characterized by similar resistance genes and mechanisms.

**Table 2:** Antimicrobial susceptibility of strain LzhVag01 and TR1180

		*D. lacustris* strain LzhVag01	*D. tsuruhatensis* strain TR1180
Antibiotic class	Antibiotics tested	MIC (mg/L)/interpretation	MIC (mg/L)/interpretation
Nitroimidazoles	Metronidazole	> 128 (R)	-
Lincosamides	Clindamycin	8 (R)	-
Aminoglycosides	Gentamicin	16 (R)	> 1024 (R)
β-Lactams	Cefoxitin	0.25 (S)	4 (S)
	Ceftazidime	0.125 (S)	0.125 (S)
	Ceftriaxone	1 (S)	8 (S)
Fluoroquinolones	Levofloxacin	2 (R)	2 (R)
Macrolides	Azithromycin	8 (S)	16 (S)
Tetracyclines	Minocycline	0.125 (S)	-
	Tetracycline	-	16 (R)

‘‘-’’ represent not tested

To get further insight into the resistance characteristics of *Delftia* isolated from human. The antimicrobial resistance (AMR) genes of LzhVag01 and TR1180 were predicted using the Comprehensive Antibiotic Resistance Database (CARD). The antibiotic resistance annotation showed that LzhVag01 contained 16 antimicrobial genes [*ceoB*, *rpsJ*, *OXA-3*, *OqxB*, *rpsL*, *thyA*, *rpoB*, *fabI*, *fusA* and other multidrug resistance genes], whereas, TR1180 including 17 antimicrobial genes [*aac(6′)-Ib3*, *aadA2*, *bla*_OXA-118_, *OqxB*, *tet(G)*, *floR*, *2sul1*, *dfrA16* and other multidrug resistance genes] (Table 3). Most of the resistance mechanisms of these resistance genes were associated with antibiotic efflux, antibiotic target alteration, antibiotic target protection, antibiotic target replacement, and antibiotic inactivation.

**Table 3:** Antibiotic resistance genes identified in the genomes of LzhVag01 and TR1180

Classification of resistance genes	*D. lacustris* strain LzhVag01	*D. tsuruhatensis* strain TR1180	Resistance mechanism
Aminoglycoside	*ceoB*, *rpsL*	*aac(6′)-Ib3*, *aadA2*	Antibiotic efflux, antibiotic target alteration, antibiotic inactivation
β-Lactam	*OXA-3*	*bla*_OXA-118_	Antibiotic target alteration
Fluoroquinolone	*OqxB*	*OqxB*	Antibiotic efflux
Tetracycline	*rpsJ*	*tet(G)*	Antibiotic target protection, antibiotic efflux
Para-aminosalicylic acid	*thyA*	*-*	Antibiotic target alteration
Rifampicin	*rpoB*	*-*	Antibiotic target alteration, antibiotic target replacement
Isoniazid-like antibiotic	*fabI*	*-*	Antibiotic target alteration
Fusidane	*fusA*	*-*	Antibiotic target alteration
Phenicol	*-*	*floR*	Antibiotic efflux
Sulphonamide	-	*2sul1*	Antibiotic target replacement
Trimethoprim	-	*dfrA16*	Antibiotic target replacement
Multidrug	*adeB*, *MuxB*, *MexD*, *soxR*, *OprJ*, *mdtC*, *abeS*	*abeS*; *mexC*, *mexD*, *oprJ*, *adeB*, *soxR*, *mdtC*, *muxB*	Antibiotic efflux

‘‘-’’ represent not found

### Distribution of Potential Virulence-Associated Genes

The existence and expression of a pathogen virulence factor (VFs) determines its capability to live, infect, and cause disease in the host. By contrasting the genomes of LzhVag01 with the VFDB, we found that LzhVag01 genome contained 11 potential virulence genes *flhA*, *motA*, *flip*, *pvdF*, *bauD*, *HsiC1/vipB/tssC*, *pilU*, *tapT*, *sugC*, *narH*, and *gspE*, which encode 8 virulence factors including Flagella, Pyoverdine, HSI-1, Type IV pili transporter, Trehalose-recycling ABC, Nitrate reductase and Gsp, respectively (Table 4). These virulence factors mainly relate to bacterial motility and adherence, iron acquisition, metabolism and secretion. Strain TR1180 included 24 putative virulence that were assumed to be associated with bacterial adhesion, biofilm formation, and stress resistance. In contrast, LzhVag01 exhibited a higher abundance of virulence factors linked to sugar metabolism and metabolic adaptation.

**Table 4:** Virulence genes identified in the genomes of LzhVag01 and TR1180

Classification of Virulence factor	*D. lacustris* strain LzhVag01	*D. tsuruhatensis strain* TR1180	Function
Flagella (offensive virulence factors)	*flhA*, *motA*, *fliP*	*cheY*, *cheW*, *flgC*, *flgG*, *fliI*, *fliP*	Motility
Pyoverdine (nonspecific virulence factor)	*pvdF*, *bauD*	*bauC*, *bauD*, *fepC*, *pvdF*	Iron uptake system
HSI-1 (offensive virulence factors)	*HsiC1/vipB/tssC*	*clpV1*, *hsiC1/vipB*, *hsiB1/vipA*	Type VI secretion system
Type IV pili (offensive virulence factors)	*pilU*, *tapT*	*pilG*, *pilT*, *pilT2*	Type IV pili
Trehalose-recycling ABC transporter (nonspecific virulence factor)	*sugC*	*-*	Sugar metabolism
Nitrate reductase (defensive virulence factors)	*narH*	*-*	Metabolic adaptation
Gsp (offensive virulence factors)	*gspE*	*-*	Type II secretion system
AdeFGH efflux pump (offensive virulence factors)	*-*	*adeG*	Biofilm
LPS (offensive virulence factors)	*-*	*acpXL*	Immune modulation
Hsp60 (offensive virulence factors)	*-*	*htpB*	Adherence
Acid resistance (defensive virulence factors)	*-*	*ureB*, *ureG*	Stress survival
Stress protein (nonspecific virulence factor)	*-*	*sodB*, *clpP*, *katA*	Stress survival

## Discussion

With the development of next-generation sequencing technology, many studies have revealed that *Delftia* was associated with a range of obstetrics and gynecology diseases, including high discomfort or pain following vaginal intercourse [15], unexplained recurrent pregnancy loss [16] and cervical cancer [14], all of which have a detrimental effect on women’s physical and emotional health. Since the *Delftia* species have yet to be successfully isolated from female vagina, nothing is known about its genomic characteristics, drug resistance characteristics, or relationships to diseases. This study isolated a *D. lacustris* strain LzhVag01 from the vaginal discharge of a 43-year-old female with CIN III in China. Antimicrobial susceptibility test and genome sequence analysis showed that the strain was multidrug-resistant and presented virulence factors. These pathogenic factors may contribute to bacterial colonization and adaptation of the vaginal environment, which accelerating the progression of cervical cancer. This study is critical to understanding the pathogenic mechanism of *Delftia lacstris* and the diagnosis and treatment of gynecological diseases.

Despite only a few cases of *Delftia* infection reported in humans, the organism has been more frequently isolated from patients with immunological deficiency in recent years. There are three recognized *Delftia* species associated with human infections: *D. acidovorans*, *D. tsuruhatensis*, and *D. lacustris* [36]. LzhVag01 was identified as *D. lacustris*, according to the phylogenetic analysis (Fig. 2). ANI analysis revealed that it had similarities with *D. lacustris* HQS1 (99.85%) and FDAARGOS 890 (98.32%). *D. lacustris* is a novel species found in freshwater in Denmark [4], and it has been isolated from samples of renal damage, hepatocellular cancer, renal infarction, and empyema in recent years [36]. Wu et al. first proposed that *Delftia* was highly enriched in the vagina of HSIL and LSIL patients and may serve as a marker for cervical cancer progression [14]. Unfortunately, they did not isolate the pathogens. Indeed, in our previous investigation assessing variations in the vaginal and cervical microbiome in women with high-risk HPV infection [13], we found that *Delftia* was discovered to be 4.6 times greater in the vagina of women with cervical cancer than in the general population, and it was shown to be twice as common in groups at high-risk of HPV. However, since *Delftia*’*s* microbial load was not too high in the vagina, it did not receive our attention at that time.

*D. lacustris* primarily inhabit freshwater environments. Initially, we thought that the patient might have become infected by contaminated water in the wild. However, the patient denied coming into contact with filthy sewage or epidemic areas, suggesting that LzhVag01 may be an opportunistic pathogen settled in the vagina. *Lactobacilli* produce lactic acid, which helps to maintain an acidic pH in the vagina. Interestingly, previous research has shown that organic acids including formic acid, lactic acid and pyruvic acid could be favorite carbon sources for the acidophilic bacteria strains of the *Delftia* species [37]. The acidified vagina environment was highly advantageous to the colonization and growth of the *Delftia* strains. To the best of our knowledge, *D. lacu*stris strain LzhVag01 was the the first strain of *Delftia* isolated from human vaginal discharge.

The *Delftia* species have a small core genome (29.5%), leading to a vast accessory genome and strain-specific genes. The number of strain-specific genes varies widely among different strains, indicating diverse genetic evolution. In LzhVag01, most strain-specific genes involved in transcription regulation, replication, recombination, and repair, with many functions remaining unknown. Further analysis was needed to clarify their roles. The pan-genome is open and evolving, suggesting that *Delftia* species can adapt to different environments and exchange genetic material. Comparative genomic analysis of three *Delftia* strains from human and natural environments revealed minor variations in genome size, with the genomes closely resembling the alignment reference genome of strain LzhVag01.

Many studies have revealed that while the pharmacological susceptibility of individual species of *Delftia* species varies widely, they are generally naturally resistant to aminoglycosides [38], and certain *D. tsuruhatensis* strains are resistant to fluoroquinolones [36]. Based on our antimicrobial susceptibility test, LzhVag01 exhibited resistance against fluoroquinolones, lincomines, nitroimidazoles, and aminoglycosides but sensitivity to β-lactams, macrolides, and tetracyclines (Table 2), which is essentially in line with the *D. tsuruhatensis strain* TR1180, a strain from the sputum of a 91-year-old female patient suffering from respiratory failure at Lishui Central Hospital in China [39]. The number of resistance genes in most *Delftia* species ranges from 0 to 20 [1], and our sequencing data revealed that LzhVag01 has 16 resistance genes in total (Table 3). With a sequencing depth of 225.68X, there was no evidence of plasmids, indicating that nearly all of these genes for resistance to antibiotics originated from the bacterial genome. The majority of *D. tsuruhatensis* species have resistance genes like *OqxB* and *MexD* [39], indicating that *Delftia* species may share these resistance genes. Notably, LzhVag01 harbors the β-lactams resistance gene *OXA-3* and the tetracyclines resistance gene *rpsJ*, yet it displays sensitivity to both β-lactams and tetracyclines, suggesting a possible lack of functionality of these genes. Additionally, strain LzhVag01 and TR1180 also harbor some strain-specific resistance genes and further verification was needed to confirm whether these genes are functional. In summary, the two strains of *Delftia* isolated from human hosts demonstrate almost identical drug-resistant characteristics and exhibit similar patterns of drug-resistant phenotypes. This contributes significantly to our comprehension about the evolution of *Delftia* resistance and furnishes vital insights for devising more efficacious antibiotic treatment approaches in the future.

Virulence factors are key factors in the infection and disease caused by bacteria, and the discovery of virulence genes can help to gain insight into the pathogenicity of bacteria and the mechanisms of interaction with hosts. We found that LzhVag01 has 11 virulence genes in total, with 63.6% (7/11) of those genes being offensive virulence factors connected to the motility, adhesion, and secretion systems of the bacterium. Compared with TR1180, LzhVag01 contained more virulence factors related to sugar metabolism and metabolic adaptation. This suggests that the variations of diverse virulence factors between these two strains may indicate their distinct adaptation strategies to various environments. Such differences aid in comprehending how bacteria survive in diverse host or environments. *PvdF* and *bauD* are linked to bacterial iron absorption. High-affinity iron absorption is exhibited by Pyoverdine, a bacterial siderophore encoded by *PvdF*. It not only efficiently obtains iron from lactoferrin and transferrin but also increases the generation of reactive oxygen species [40]. Importantly, reactive oxygen species can cause double-stranded DNA breaks in both the host and HPV genomes [41], allowing HPV to integrate into the host genome and causing carcinogenesis. The enzyme encoded by *narH* can increase the expression of nitrate reductase in hypoxic conditions in *Mycobacterium tuberculosis* (*Mycobacterium gilvum*), which in turn increases nitrate reduction [42]. This process helps bacteria survive in hypoxic inflammatory or necrotic tissues. Indeed, our clinical data also showed that the vaginal cleanliness of this 43-year-old female was degree III, which means abnormal vaginal cleanliness with inflammation. Therefore, we speculate that the *narH* may be related to adaptation to the hypoxic environment of the vagina. Lastly, a variety of virulence factors found in LzhVag01 help the bacteria colonizing in the vagina and adapt to its environment. In conclusion, in addition to helping the bacteria colonize the vagina and adapt to its surroundings, LzhVag01 carried a variety of virulence characteristics that could promote the onset of cervical cancer.

This study analyzed the whole-genome sequence of *D. lacustris* strain LzhVag01 isolated from vaginal discharge, which will provide support for further understanding of the molecular pathogenesis of *Delftia*. More investigation is required to confirm whether the immune suppression of cervical cancer or other factors contributes significantly to the enrichment of *Delftia.* Nevertheless, our study demonstrates that the presence of multiple pathogenic factors in LzhVag01 may accelerate the progression of cervical cancer. Therefore, it is recommended that, in addition to the treatment of cervical cancer, factors such as abnormal vaginal microbiota, *Delftia* enrichment, and antibiotic use should be taken into account.
